# Advancing Sclerotinia risk forecasting for winter rapeseed in Germany: integrating crop phenology and disease development into a decision support system

**DOI:** 10.1002/ps.70166

**Published:** 2025-08-22

**Authors:** Vera Krause, Nazanin Zamani‐Noor, Lena Müller, Hella Kehlenbeck, Anto Raja Dominic

**Affiliations:** ^1^ Julius Kühn‐Institute (JKI), Institute for Strategies and Technology Assessment Kleinmachnow Germany; ^2^ Julius Kühn‐Institute (JKI), Institute for Plant Protection in Field Crops and Grassland Braunschweig Germany; ^3^ Central Institute for Decision Support Systems in Crop Protection (ZEPP) Bad Kreuznach Germany

**Keywords:** Sclerotinia stem rot, *Sclerotinia sclerotiorum*, *Brassica napus*, integrated pest management (IPM), SkleroPro, pest risk

## Abstract

**BACKGROUND:**

Sclerotinia stem rot, caused by *Sclerotinia sclerotiorum*, threatens winter rapeseed (*Brassica napus*) production in Germany, with potential yield losses of up to 30%. The current SkleroPro model provides regional Sclerotinia risk assessments but has shown declining predictive accuracy. This study aims to enhance SkleroPro by integrating a newly developed phenological model to predict flowering stages and a sclerotia germination module to improve disease risk forecasting.

**RESULTS:**

A phenological model was developed using temperature and photoperiod as key predictors. The model achieved a root mean square error (RMSE) of 3.83 days for predicting flowering stages (BBCH 58–70). A sclerotia germination model was created, with 79% accuracy, incorporating mean maximum temperature and relative humidity as predictors. Integration of these models into SkleroPro improved disease risk prediction, increasing accuracy from 39% to 66%. Sensitivity rose to 90%, ensuring a low risk of underestimating disease outbreaks.

**CONCLUSION:**

The enhanced SkleroPro model improves disease risk forecasting by identifying high‐ and low‐risk windows for fungicide application, reducing unnecessary treatments while maintaining effective disease control. This decision support tool promotes sustainable winter rapeseed production. The model is currently undergoing further validation with the German Plant Protection Services before being made freely available to farmers. © 2025 The Author(s). *Pest Management Science* published by John Wiley & Sons Ltd on behalf of Society of Chemical Industry.

## Graphical abstract

Integrating crop phenology and sclerotia germination models into SkleroPro improved Sclerotinia risk prediction accuracy from 34% to 66%, optimizing fungicide timing and supporting sustainable winter rapeseed production.

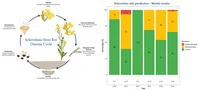

## 1 INTRODUCTION

Winter rapeseed (*Brassica napus* L.) is a widely cultivated oilseed crop across most European countries, and its growth is influenced by a complex interplay of factors.^1^, ^2^, ^3^ Key determinants that influence its development include the cultivar's genetic traits, weather conditions, and the prevalence of pests and diseases.^4^ Sclerotinia stem rot, caused by the phytopathogen *Sclerotinia sclerotiorum* (Lib.) de Bary, is a significant disease affecting various crops, including winter rapeseed. In Germany, the incidence of Sclerotinia stem rot can result in yield losses of up to 30%.^5^ This pathogen is well known for its extensive host range and its ability to persist in soil as sclerotia for several years, making it a persistent threat to agricultural productivity.^6^ Under suitable conditions, sclerotia germinate forming apothecia, which produce ascospores. Following successful infection of aerial plant parts by the airborne ascospores, the pathogen colonizes plant tissue, causing premature flowering and wilting, leading to a significant reduction in crop yield.^7^, ^8^, ^9^ Disease progression is influenced not only by weather variables^6^, ^10^ but also by differences in aggressiveness among pathogen isolates, which can significantly affect the occurrence and development of Sclerotinia stem rot.^11^ A single fungicide application is typically recommended during early flowering, as infection usually begins at petal fall when petals provide nutrient‐rich sites for germinating ascospores,^12^, ^13^ though root infections may also occur.^14^

Decision support systems (DSSs) are valuable tools in modern agriculture, providing farmers and agronomists with important insights to make timely decisions.^15^, ^16^, ^17^ These systems integrate diverse data sources, including weather data, information about crop and field management practices and landscape data to simulate crop and disease development and perform pest risk assessments. The adoption of DSSs can significantly enhance the efficiency of crop management practices, reduce dependency on chemical inputs, and mitigate the impact of diseases like Sclerotinia stem rot.^10^, ^16^, ^18^ Because fungicide applications must be made before symptoms appear, accurate timing is essential, underscoring the importance of reliable forecasting models for effective Sclerotinia management.^19^

Phenological models, which are integral components of crop growth models, predict the timing of key developmental stages and are critical for scheduling monitoring, fertilization and pest control measures.^20^, ^21^ They ensure that protective measures are applied during the most vulnerable stages of the crop, thereby enhancing overall management effectiveness.^22^, ^23^ Several models have been developed describing the effect of temperature and photoperiod on the phenological development of rapeseed.^1^, ^3^, ^4^, ^24^, ^25^ Despite the availability of phenological models for winter rapeseed in Germany,^10^, ^21^, ^26^ there are notable deficiencies: the data used for these models date back to the late 1990s and early 2000s. Due to changing climate conditions, Simonto‐WR,^27^, ^28^ widely used for predicting flowering stages, has shown a decline in predictive accuracy in recent years. In addition, Simonto‐WR also requires the user to input the date of BBCH 55 growth stage, which may not always be feasible in practice due to time, labor, or access constraints.

While modeling crop phenology is required for the identification of vulnerable crop stages, developing models for the various stages of the life cycle of *S. sclerotiorum* is equally important to predict the pathogen's critical periods of activity. These models can predict the germination of sclerotia and the subsequent release of ascospores, which are responsible for initiating infections and serve as the primary inoculum source.^29^, ^30^ The germination of sclerotia of *S. sclerotiorum* is influenced by environmental factors such as temperature, humidity, and soil moisture, with cool, moist conditions promoting the development of apothecia and release of ascospores.^6^, ^30^

Clarkson *et al*.^6^ developed a model for *S. sclerotiorum* infection and disease development on lettuce quantifying the effects of temperature, relative humidity and ascospore density. The weather‐based model of Willbur *et al*.^31^ was developed to assess the risk of Sclerotinia apothecial presence in soybean whereas that of Salotti and Rossi^19^ predicts Sclerotinia disease progress on soybean and sunflower. However, forecasting diseases caused by *S. sclerotiorum* remains challenging, as existing literature often shows inconsistent associations between environmental and agronomic factors and the various life stages of the pathogen.^29^ Koch *et al*.^10^ developed a forecasting model named SkleroPro, which provides a regional assessment of the Sclerotinia stem rot risk and recommendations for fungicide application based on a cost–benefit analysis in Germany. This model calculates infection hours critical for infection, defined by temperature and relative humidity conditions in the plant canopy. However, the accuracy of this model has declined in the recent years, limiting its practical use (ZEPP and ISIP, 2019, pers. comm.). This decline likely stems from a biological limitation in the model – sclerotia germination is not considered, leading to possible overestimations when conditions favored infection but viable inoculum was absent. Assuming uniform inoculum presence across fields, especially those with low or absent sclerotial populations, may not reflect field reality and contribute to reduced model reliability.

Therefore, developing a more reliable phenological model and improving Sclerotinia disease risk forecasting are crucial for effective disease management. Our study aims to advance the current SkleroPro model to support informed fungicide application decisions by identifying high‐ and low‐risk windows for Sclerotinia stem rot during the flowering period of winter rapeseed in Germany. We have addressed this concern by:

- Developing a new phenological model to predict flowering stages (BBCH 58–70);
- Integrating the newly developed phenological model into an improved version of the SkleroPro forecasting system;
- Designing a new module within SkleroPro to predict sclerotia germination and ascospore release;
- Validating the new phenological model and the sclerotia germination model using field data;
- Assessing the performance of the enhanced SkleroPro model against disease monitoring data from 2020 to 2024.

## 2 MATERIALS AND METHODS

### 2.1 Data sources and preparation

#### 2.1.1 Phenology data

We used phenology data recorded in the BBCH code scale^32^ for 1651 site‐year combinations across Germany during the period 2020–2024 (Fig. 1). The dataset consisted of growth stages from BBCH 50 to BBCH 80 along with the coordinates, cultivar and sowing date. The data were sourced from the ISIP (German Information System for Integrated Plant Production) monitoring platform and field trial observations provided by the Plant Protection Services from 12 German federal states.^33^ The data collected under practical field conditions resulted in a total of 10 539 observations (1397 from the field trials and 9142 from the monitoring data). Growth stages were assessed visually every 7 days, recording the predominant BBCH stage in the field. For 142 of these site‐years during the period from 2020 to 2023, 25 plants per field were assessed, resulting in 25 BBCH values per field.

**Figure 1:**
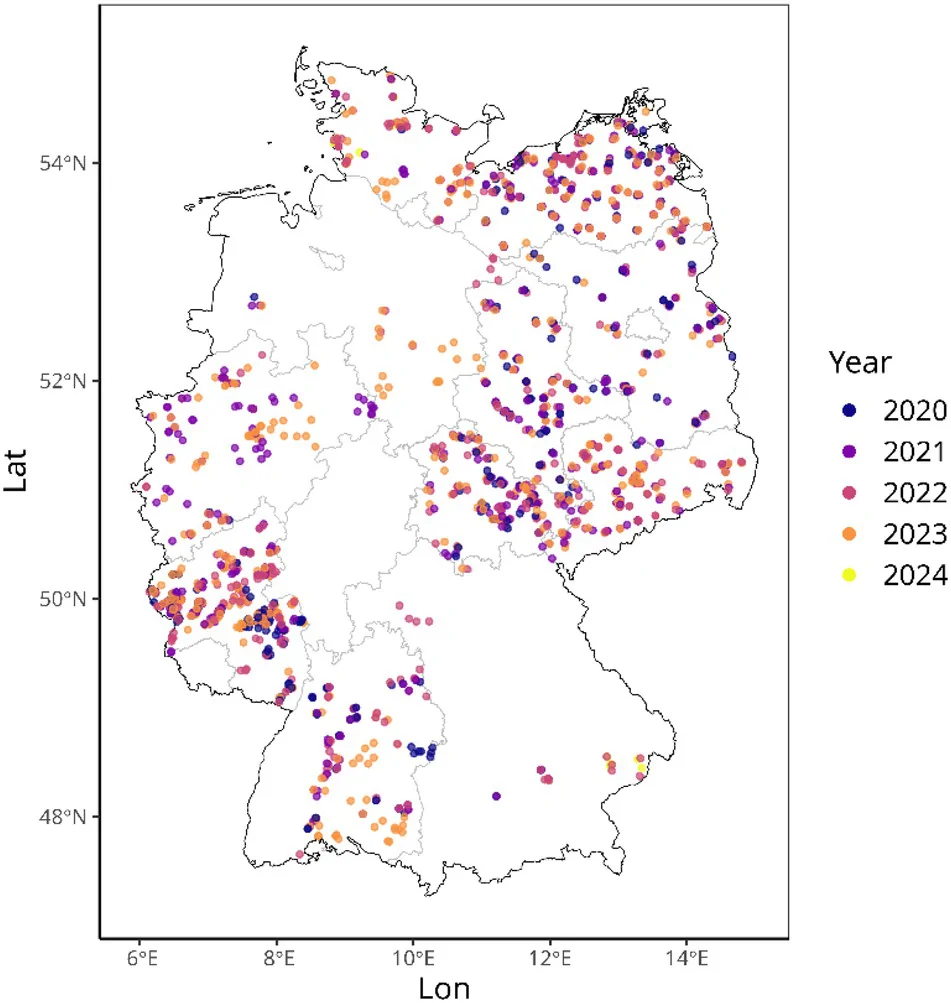
Map of Germany showing the BBCH monitoring sites (dots) for the period 2020–2024. Each color represents a different year. Lat, latitude (°N); Lon, longitude (°E).

#### 2.1.2 Sclerotinia disease incidence monitoring data

Sclerotinia disease incidence (DI) data were assessed and collected at plant growth stages 80–83 by seven German federal states between 2020 and 2024 (Fig. 2), covering a total of 37 site‐years (Supporting Information Table S1). DI was measured as the percentage of infected plants in untreated field trials, focusing on lesions present on the main stem, which are typically associated with significant yield loss. In each trial, 100 plants were assessed, with 25 plants examined per replicate across four replicates. In some trials, two different cultivars were tested. Cultivar effects were excluded from the analysis, as no site showed evidence of tolerance to Sclerotinia infection.

**Figure 2:**
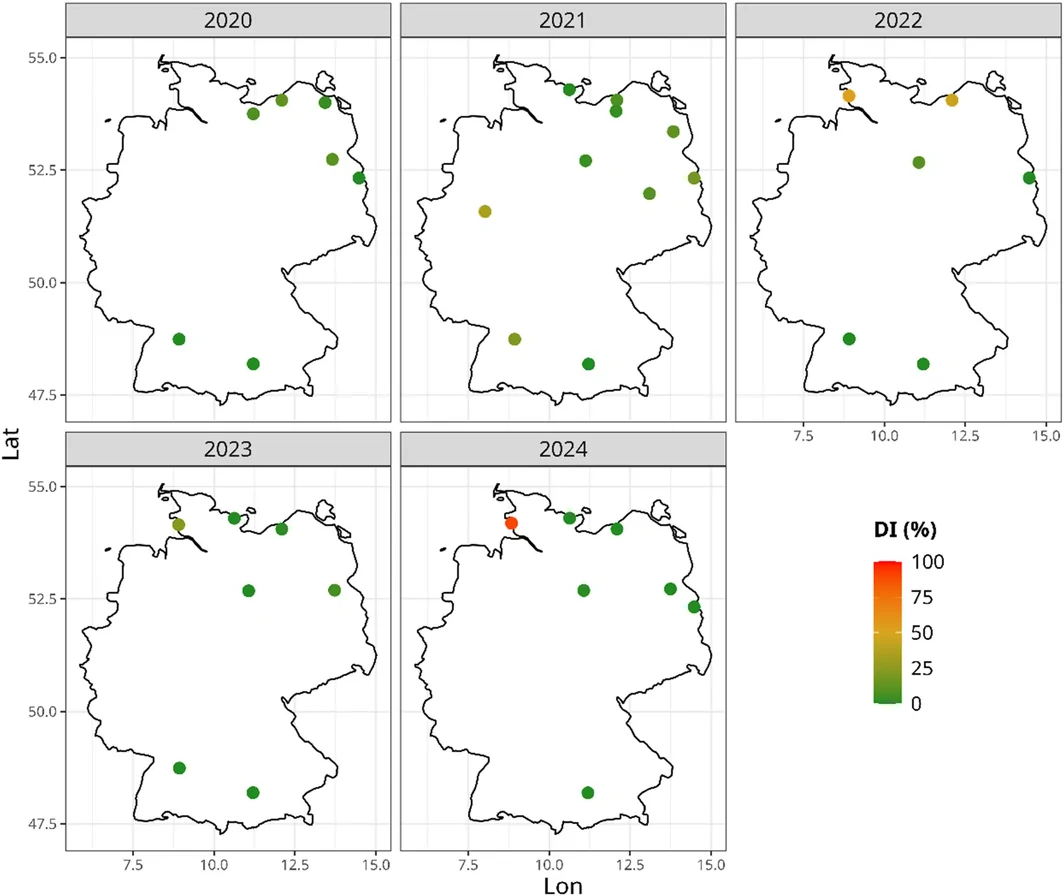
Sclerotinia disease incidence (DI) data collected by seven German federal states between 2020 and 2024 from a total of 37 site‐years. Each panel represents a year and each dot represents a site. The colors represent the Sclerotinia DI (%), ranging from high incidence (red) to low (green). Lat, latitude (°N); Lon, longitude (°E).

#### 2.1.3 Sclerotia germination experiments

To examine the effects of weather on sclerotia germination and apothecia development, four depots (each with 100 sclerotia) were established annually in late October from 2020 to 2023 near the experimental winter rapeseed field on the Julius Kühn‐Institute (JKI) campus in Brunswick, Germany.^11^ A depot is a field site where sclerotia are buried to monitor apothecia formation. Spaced ~500 m apart, depots used native soil without amendments. Sclerotia were buried at 3 cm depth and left undisturbed. Apothecia emergence was monitored from April to mid‐June, with 2–3 observations per week. Environmental data – soil temperature and rainfall – were recorded every 30 min using a weather data logger (Tinytag Plus 2‐TGP‐4020; Gemini Data Loggers Ltd, Chichester, UK).

#### 2.1.4 Weather data

We obtained daily weather data from the German Weather Service (DWD),^34^ interpolated over a 1 km × 1 km grid. The parameters used for the study consisted of daily mean, minimum and maximum air and soil temperature (*T*_mean_, *T*_min_, *T*_max_, °C), precipitation sum (in millimeters) and relative humidity (%).

### 2.2 Model development and validation

#### 2.2.1 Phenological model

To predict phenological stages between BBCH 58 and BBCH 70, we developed a new phenology model based on the approach by Soltani and Sinclair^35^ using daily mean air temperature (°C) and photoperiod (in hours) for each site starting from 1 February of each year. We calculated the photoperiod using R function ‘daylength’ from package ‘Geosphere’, which computes day length based on latitude and date.^36^

For each site, we calculated a daily temperature factor tempfun, a three segment linear function with a scalar factor ranging from zero to one, representing the curvilinear plant response to temperature (Fig. 3).^37^, ^38^ This function requires four parameters: base temperature (TBD, °C), lower optimum temperature (TP1D, °C), upper optimum temperature (TP2D, °C), and ceiling temperature (TCD, °C). We calculated the daily photoperiod factor ppfun, which quantifies the development response to photoperiod as a value between zero and one, using a quadratic function adopted from the DSSAT‐CERES model.^39^ Tempfun and ppfun were calculated as in Eqns (1) and (2).

(1) $$\text{tempfun}=f\left(x\right)=\left\{\begin{array}{c} \;0,\;\text{if}\;\mathrm{TBD}\geq T\geq \mathrm{TCD}\\ \frac{T-\mathrm{TBD}}{\mathrm{TP}1D-\mathrm{TBD}},\;\text{if}\;\mathrm{TBD}<T<\mathrm{TP}1D\\ 1,\;\text{if}\;\mathrm{TP}1D\leq T\leq \mathrm{TP}2D\\ \frac{\mathrm{TCD}-T}{\mathrm{TCD}-\mathrm{TP}2D},\;\text{if}\;\mathrm{TP}2D<T<\mathrm{TCD} \end{array}\right.$$

(2) $$\text{ppfun}=\left\{\begin{array}{cc} 1-\left(\text{ppsen}\times {\left(\mathrm{CPP}-\mathrm{PP}\right)}^{2}\right), & \text{if}\;\mathrm{PP}<\mathrm{CPP}\\ 1, & \text{if}\;\mathrm{PP}\geq \mathrm{CPP} \end{array}\right.$$

where, PP is the daily photoperiod (in hours), CPP is the critical photoperiod (in hours) above which development proceeds without day length effects, and ppsen is the photoperiod sensitivity coefficient.

**Figure 3:**
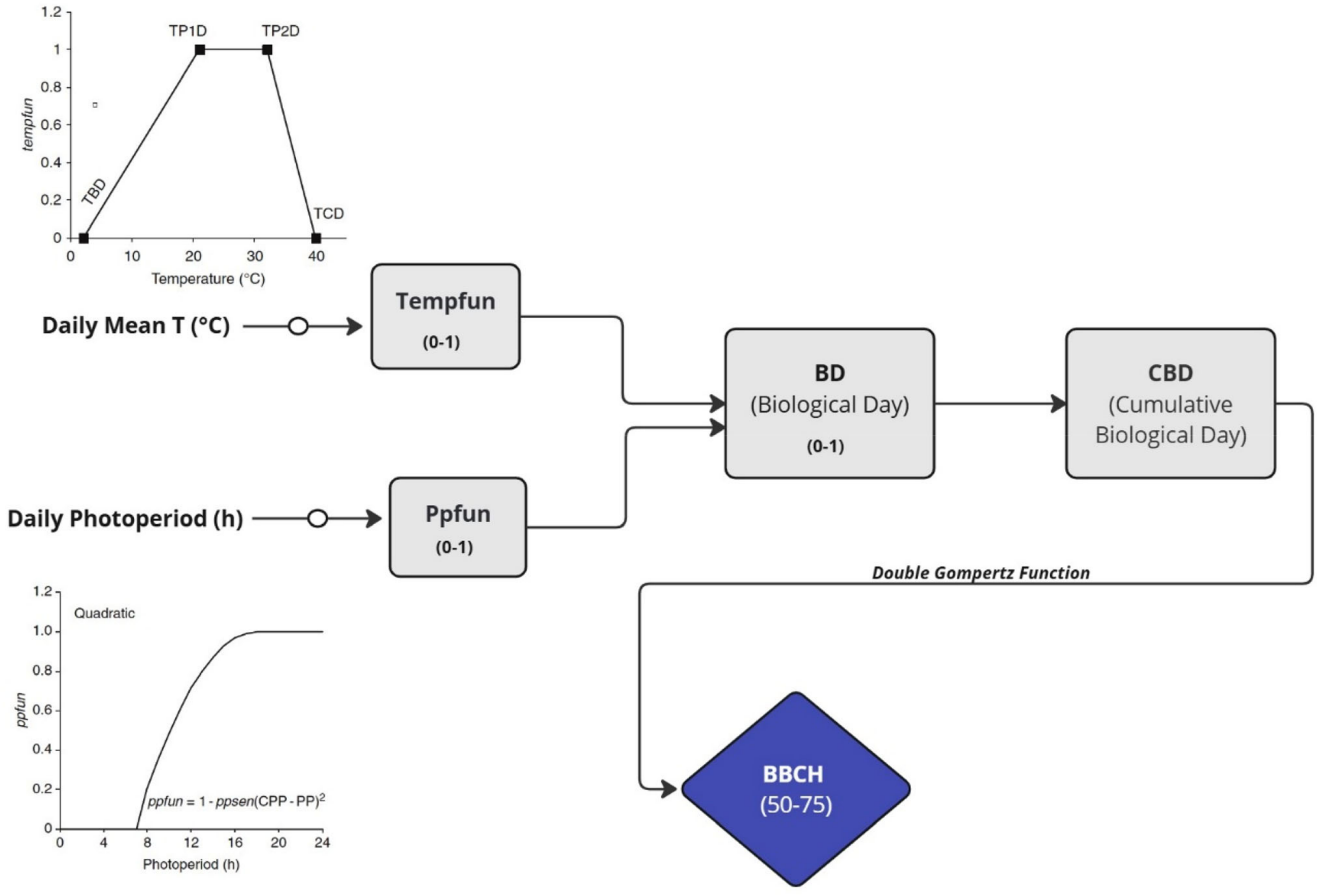
Schema for the calculation of the cumulative biological day (CBD). The insets describe the tempfun (upper) and the ppfun (lower) functions. TBD, base temperature (°C); TP1D, lower optimum temperature (°C); TP2D, upper optimum temperature (°C); TCD, ceiling temperature (°C); PP, daily photoperiod (hours); CPP, critical photoperiod (hours); ppsen, photoperiod sensitivity coefficient; Daily Mean T (°C), daily mean temperature (°C); Daily Photoperiod (h), daily photoperiod (hours).

We calculated the daily biological day (BD) as the product of tempfun and ppfun (Eqn (3)), which was then summed up daily to provide the cumulative biological day (CBD, Eqn (4)).

(3) BD=tempfun×ppfun

(4) $${\mathrm{CBD}}_{i}={\mathrm{CBD}}_{i-1}+{\mathrm{BD}}_{i}$$

where CBD for day *i* is the sum of the daily BD and the CBD from day *i* − 1. The product approach (Eqn (3)) accounts for the inhibition of development due to suboptimal temperatures and/or photoperiod conditions.

We transformed CBD values into BBCH by using a double Gompertz function (Eqn (5)) with four adjustable parameters:

(5) $$\text{BBCH}=b+\left(c-b\right)e^{-e^{a\left(\mathrm{CBD}-d\right)}}$$

where BBCH = calculated BBCH stage, and *a*, *b*, *c* and *d* are function constants which are different for the two Gompertz functions. The use of two Gompertz functions was intended to reflect the distinct growth phases during phenological development.^26^ The first function (for CBD ≤ 22.5) models the rapid progression from BBCH 58 to BBCH 65 (beginning of flowering to full bloom), while the second function (for CBD > 22.5) captures the slower development from BBCH 65 to BBCH 75 (full bloom to end of flowering) (Supporting Information Fig. S1).

We tested sowing date effects on flowering onset but found no significant correlation (Fig. S2), likely because flowering in winter rapeseed is mainly driven by late winter and spring conditions. The use of ~60 cultivars with differing growth dynamics may have contributed to the lack of a clear sowing date effect. Therefore, model computations began on 1 February of each year, regardless of sowing date.

We calibrated the model using BBCH data from 142 site‐years (2020–2023), based on detailed observations of 25 plants per field to ensure accuracy. In contrast, ISIP monitoring data (2020–2024), primarily collected for pest monitoring, included BBCH stages estimated informally during routine observations. Consequently, trial data were used for calibration, and ISIP data for validation. Model parameters (TBD, TP1D, TP2D, TCD, CPP) were calibrated using a grid search, systematically testing value combinations within predefined ranges. For each combination, the model was run across five‐fold cross‐validation subsets of the 142 site‐years, and root mean squared error (RMSE) was calculated between observed and simulated BBCH stages. The parameter set yielding the lowest average RMSE across folds was selected as optimal for the model. Model validation involved comparing observed and simulated BBCH stages and their corresponding dates from 1509 ISIP site‐years, focusing on flowering stages (BBCH 58–70). In addition to RMSE, we computed the mean bias error (MBE) to quantify systematic deviation and the coefficient of determination (*R* ^2^) to assess prediction accuracy.

#### 2.2.2 Sclerotia germination model

We developed a weather‐based model to predict daily sclerotia germination rates based on the germination rates recorded at the sclerotia depots. In addition to the weather parameters from the weather data loggers, we used weather parameters from the DWD.^34^ We calculated sums or averages depending on the variables for 3, 5 and 10 days before the date of observation, resulting in a total of 45 potential predictors (Table 1).

**Table 1:** List of the total weather variables and the aggregated derivatives used as potential predictors for the sclerotia germination rate model

Variable (daily)	Additional aggregates
Precipitation (mm)	Sum and average for 10, 5 and 3 days
Precipitation‐TT (mm)	Sum and average for 10, 5 and 3 days
Mean air temperature (°C)	Average for 10, 5 and 3 days
Mean air temperature (°C)	Sum for 10, 5 and 3 days (base = 5°C)
Soil temperature‐TT (°C)	Average for 10, 5 and 3 days
Soil temperature	Sum for 10, 5 and 3 days (base = 5°C)
Maximum temperature (°C)	Average for 10, 5 and 3 days
Minimum temperature (°C)	Average for 10, 5 and 3 days
Ambient relative humidity (%)	Average for 10, 5 and 3 days
Number of days when air temperature ≥ 5°C	Sum for 10, 5 and 3 days

Variables with the suffix ‘‐TT’ correspond to measurements recorded by the weather data logger, while those without the suffix represent weather data obtained from the German Weather Service (DWD).^34^ All additional aggregates were calculated over the last 10, 5, and 3 days preceding the date of observation, either as cumulative sums and/or averages. Combined with the daily variables, this resulted in a total of 45 variables, all of which are present in Fig. 8.

We applied the Boruta algorithm,^40^ a random forest‐based feature selection method^41^, ^42^, to identify key predictors of sclerotia germination. The variables deemed as ‘confirmed’ by the Boruta algorithm were further refined using stepwise regression with a generalized linear model (GLM) as implemented in the R function *stepAIC* from the R package *MASS*.^43^ We fitted a binomial logistic regression model with sclerotia germination as the outcome variable and the weather variables selected from the *stepAIC* step as predictors. We validated the model with five‐fold cross‐validation, and assessed performance by calculating accuracy, sensitivity and specificity from a confusion matrix, implemented using *caret* package.^44^

To refine the accuracy of Sclerotinia infection predictions, we recalibrated optimal temperature and relative humidity parameters through a grid search optimization using 2019–2023 field trial data. These parameters define optimal infection conditions for calculating infection hours.^10^ We tested value combinations within set ranges using five‐fold cross‐validation on 59 units (site‐cultivar), selecting the combination with the highest prediction accuracy.

#### 2.2.3 Incorporation of sclerotia germination model to the SkleroPro

We incorporated the sclerotia germination module into the existing SkleroPro model^10^ (Fig. 4). The sclerotia germination module is triggered when the phenology module predicts BBCH 58. From this stage onwards till the end of the flowering period, ascospores can infect plants via flower petals (Fig. 5). This module assumes that sclerotia germination leads to the formation of apothecia which release ascospores. Ascospores are assumed to be available for potential infection and remain viable for up to 7 days, even under suboptimal environmental conditions.^6^, ^45^ Infection hours are only calculated when viable ascospores are presumed present. The model then proceeds as in the current version to guide fungicide application based on economic damage thresholds. Additionally, user‐provided crop rotation data^10^ to help modulate infection hour thresholds; shorter rotations with susceptible crops lower the threshold, increasing infection risk by reflecting greater sclerotia presence near the soil surface. Adjacent field effects were excluded due to lack of spatial data.

**Figure 4:**
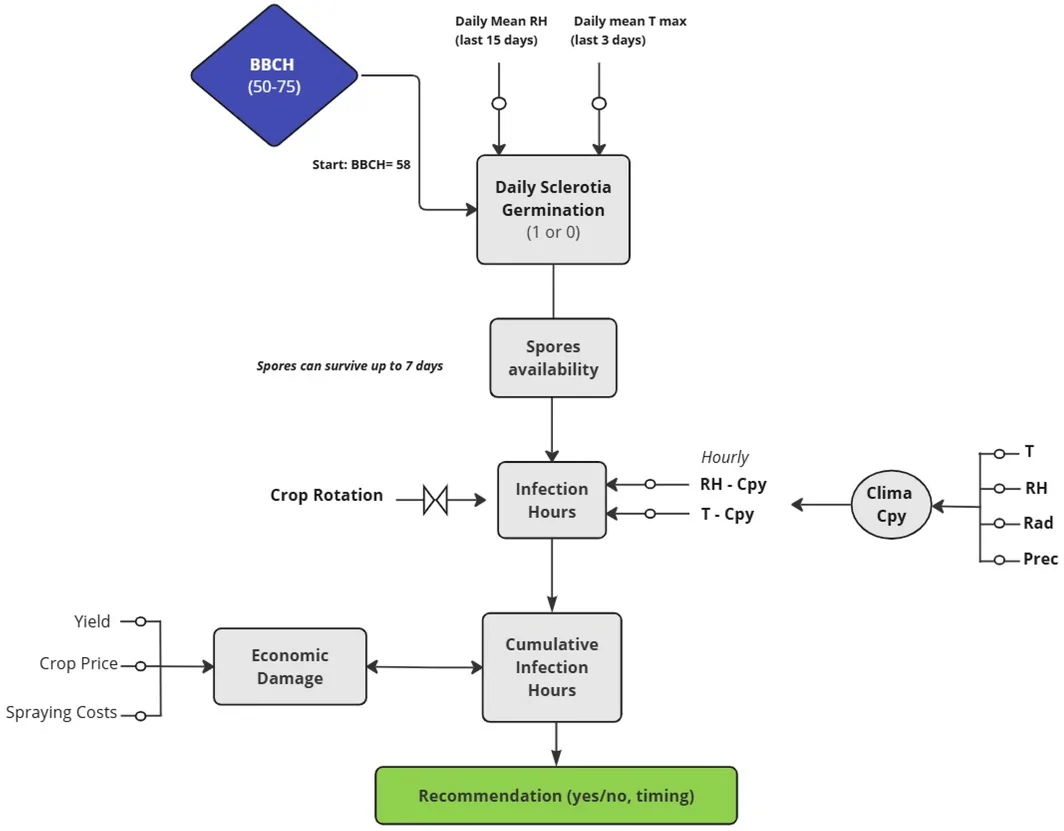
Schema of the SkleroPro model (adapted from Koch *et al*.,^10^) with the new sclerotia germination module. Daily Mean RH (last 15 days), daily mean relative humidity (%) from the previous 15 days; Daily Mean T max, daily mean maximum temperature (°C) from the last 3 days; T, hourly temperature (°C); RH, hourly relative humidity (%); Rad, daily global radiation (W/m^2^); Prec, hourly precipitation (mm); Clima Cpy, microclimate in the canopy; RH‐Cpy, relative humidity in the canopy; T‐Cpy, temperature in the canopy.

**Figure 5:**
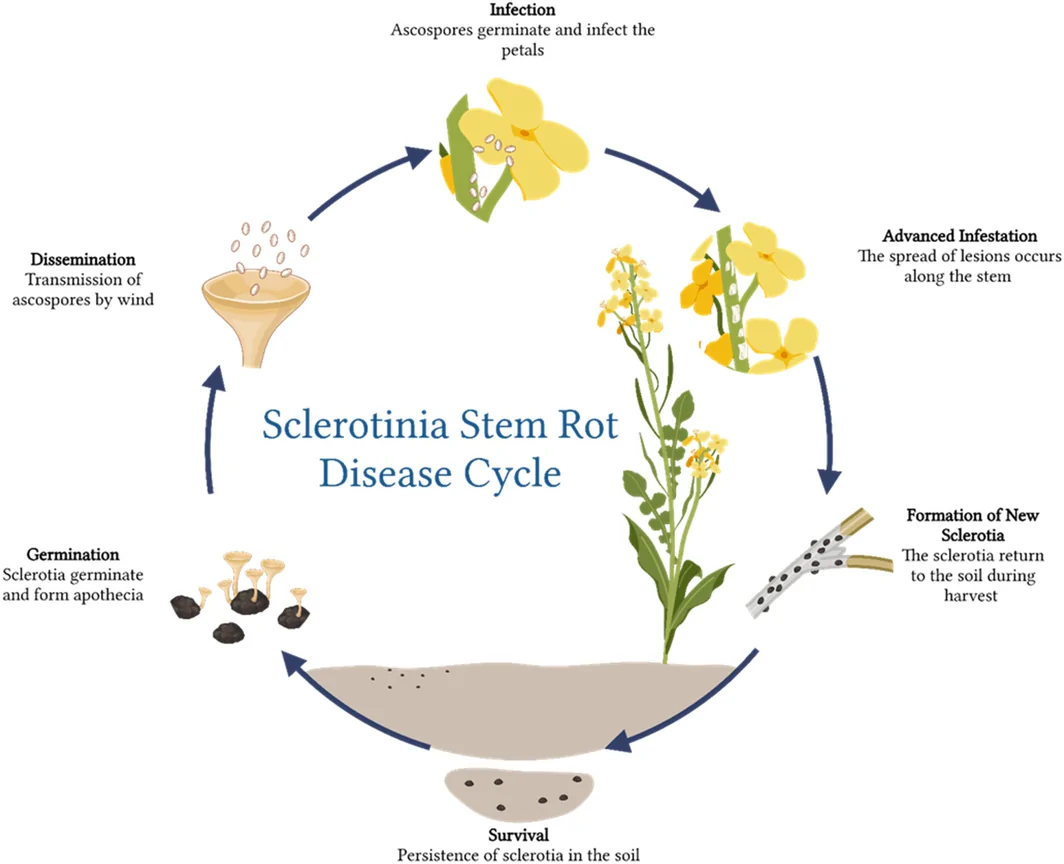
Life cycle of *Sclerotinia sclerotiorum* and infection of winter rapeseed. Created with BioRender.com.

#### 2.2.4 Validation of the improved SkleroPro

We validated the improved SkleroPro model using Sclerotinia DI data from untreated fields, treating site‐years with multiple cultivars as separate units for a total of 59 units across 37 site‐years (2019–2024). Based on consultations with German Plant Protection Services, we used a 20% DI threshold, representing the proportion of plants with visible main stem infection linked to significant yield loss. We calculated sensitivity (True Positive Rate) and specificity (True Negative Rate) from predicted and observed DI using a confusion matrix (Table 2). Treatment recommendations were considered only until the crop reached BBCH 70, as later treatments do not reduce infection risk. A model output exceeding the risk threshold indicates that environmental conditions during flowering may result in DI surpassing 20% by harvest time, potentially causing significant yield loss.

**Table 2:** The confusion matrix used for validating the new SkleroPro model.

Disease incidence (%)	Treatment recommendation (until BBCH 70)	No treatment recommendation (until BBCH 70)
≥ 20	Correct	Underestimation
<20	Overestimation	Correct

## 3 RESULTS

### 3.1 Phenology model

The CBD model achieved a RMSE of 3.83 for BBCH stages 58–70, corresponding to an average prediction error of approximately 4 days (Fig. 6). The calibrated parameters for both the CBD model and the Gompertz equation constants are presented in Tables 3 and 4.

**Figure 6:**
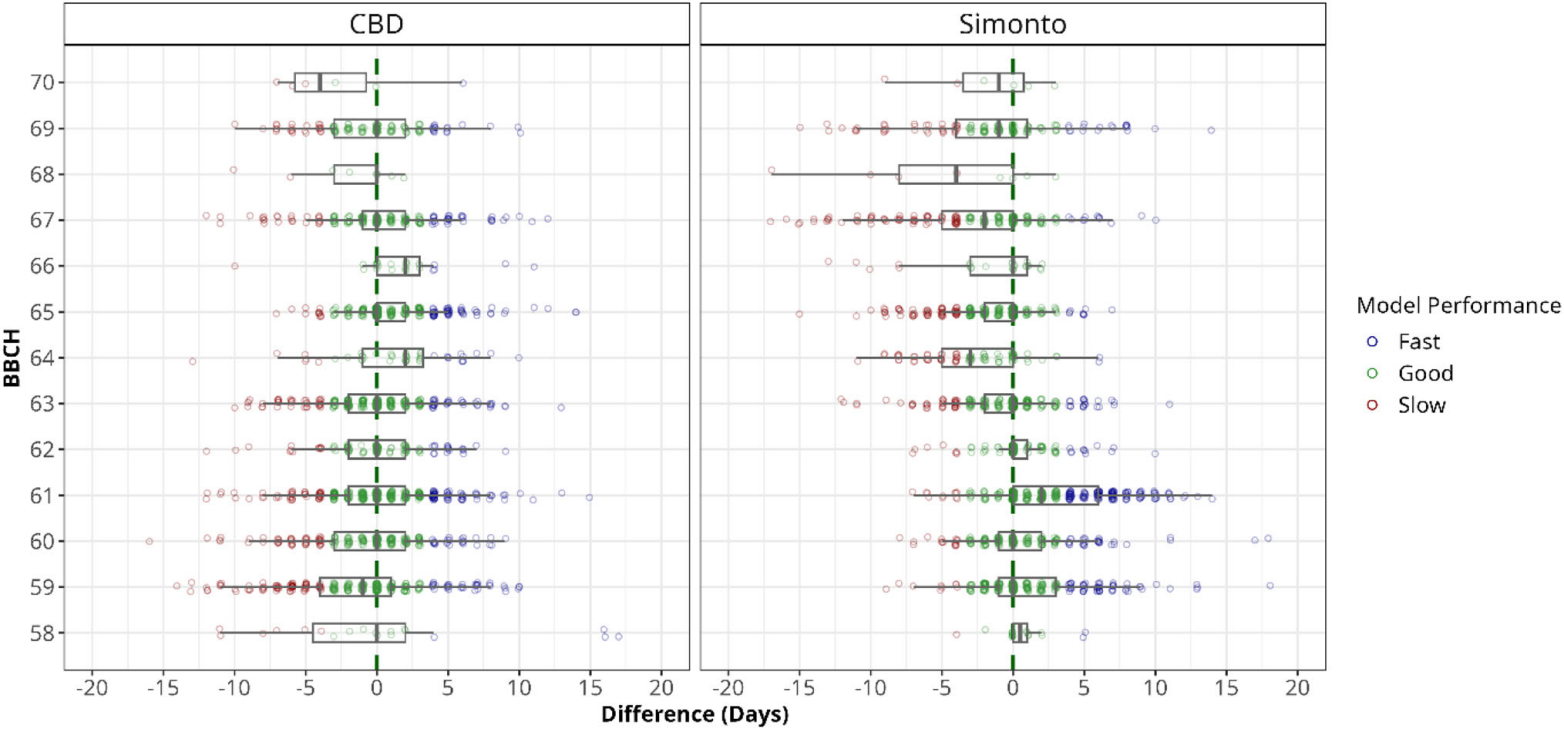
Model performance comparison between cumulative biological day (CBD) and Simonto‐WR. The *x*‐axis depicts the difference in days between the observed and predicted dates for the individual BBCH stages (*y*‐axis) for both models. Each point represents a site‐year and is categorized based on the prediction accuracy into fast (blue, > 3 days), good (green, between 3 and −3 days) and slow (red, < −3 days). The points are overlaid with box‐plots for each BBCH stage.

**Table 3:** List of parameters for the tempfun and ppfun functions used in the cumulative biological day (CBD) model and their calibrated values

Parameter	Range	Source	Calibrated
TBD (°C)	0–6	^2^, ^4^, ^21^, ^26^	0
TP1D (°C)	20–22	^3^, ^4^, ^26^	20.5
TP2D (°C)	25	^4^	25
TCD (°C)	35–40	^4^, ^26^	35
CPP (h)	11–18	^21^, ^24^, ^35^	18
ppsen	0.0021	^4^	0.0021

Abbreviations: TBD, base temperature (°C); TP1D, lower optimum temperature (°C); TP2D, upper optimum temperature (°C); TCD, ceiling temperature (°C); CPP, critical photoperiod (hours); ppsen, photoperiod sensitivity coefficient.

**Table 4:** The calibrated constants for the double Gompertz function used in the cumulative biological day (CBD) model

*a*_1_	−0.25	*b*_1_	50.66	*c*_1_	66.12	*d*_1_	15.96
*a*_2_	−0.10	*b*_2_	63.15	*c*_2_	76.22	*d*_2_	31.00

Constants with subscript 1 correspond to the first Gompertz function (CBD values ≤ 22) while constants with subscript 2 correspond to the second Gompertz function (CBD values > 22).

When comparing CBD and Simonto‐WR models, the CBD model showed a lower RMSE, higher *R* ^2^, and minimal bias (Table 5). However, the gains in accuracy were minimal. While the improvement in average error was modest, variability remained in the predictions for both models (Fig. 6). Specifically, the CBD model showed greater variability in predictions for BBCH 58 and BBCH 59. Conversely, the Simonto‐WR model exhibited a delay in predictions from BBCH 64 onwards, with simulated dates lagging behind the observed dates.

**Table 5:** Comparison of the cumulative biological day (CBD) and Simonto‐WR phenology models based on three performance metrics: root mean square error (RMSE), mean bias error (MBE) and the coefficient of determination (*R* ^2^).

Metrics	CBD	Simonto‐WR
RMSE	3.83	4.27
MBE	−0.03	−0.06
*R* ^2^	0.90	0.88

### 3.2 Sclerotia germination model

The results of the sclerotia germination experiments are presented in Fig. 7, which shows that germination primarily occurred between mid‐April and late May, coinciding with periods of increased rainfall and rising soil temperatures. The Boruta algorithm identified ten weather variables as important predictors of sclerotia germination (Fig. 8). Stepwise Akaike information criterion (AIC) refinement further reduced these to two key variables: mean daily maximum temperature over the last 3 days (tmax_3d) and mean relative humidity over the last 15 days (rhmean_15d). The logistic binomial model (Eqn (6)) classified germination probability using a 0.5 threshold (Eqn (7)). The model uses a binary classification where probabilities > 0.5 indicate sclerotia germination (1) and ≤ 0.5 indicate no germination (0). The final sclerotia germination model achieved an overall accuracy of 79% (Table 6), with a sensitivity (True Positive Rate) of 85% and a specificity (True Negative Rate) of 71%. Underestimation occurred in 9% of cases (False Negative Rate). The calibrated parameters for optimal temperature and optimal relative humidity for Sclerotinia infection are presented in Table 7.

(6) $$\text{Germination Probability}=\frac{e^{-36.74-0.33\times t\max\_3d+0.62\times r\text{hmean}\_15d}}{1+e^{-36.74-0.33\times t\max\_3d+0.62\times r\text{hmean}\_15d}}$$

(7) $$\text{Germination}=\left\{\begin{array}{c} 1,\text{if Germination Probability}>0.5\\ 0,\text{if Germination Probability}\leq 0.5 \end{array}\right\}$$

**Figure 7:**
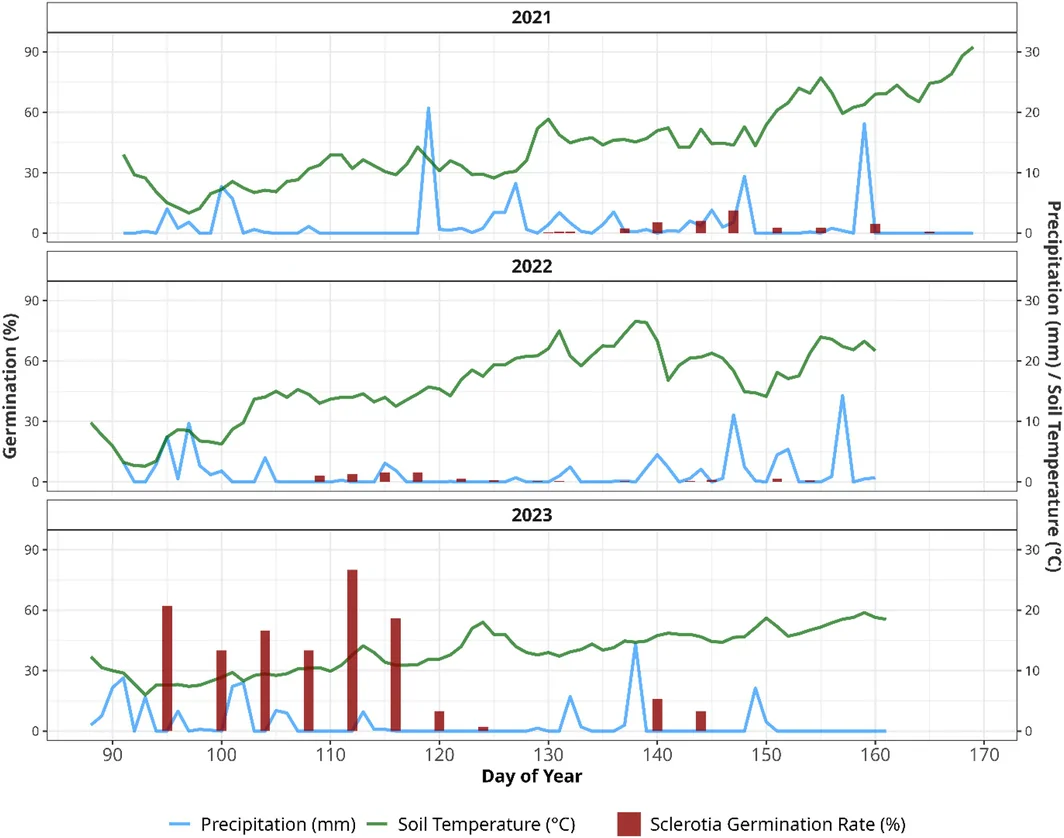
Field observations of sclerotia germination over 3 years: 2021, 2022, and 2023. Dark red bars indicate the sclerotia germination rate (%), while the blue and green lines represent daily precipitation (mm) and soil temperature (°C), respectively, plotted by day of year (DOY) from late March to mid‐June. Precipitation and soil temperature were recorded daily, whereas sclerotia germination was assessed two to three times per week.

**Figure 8:**
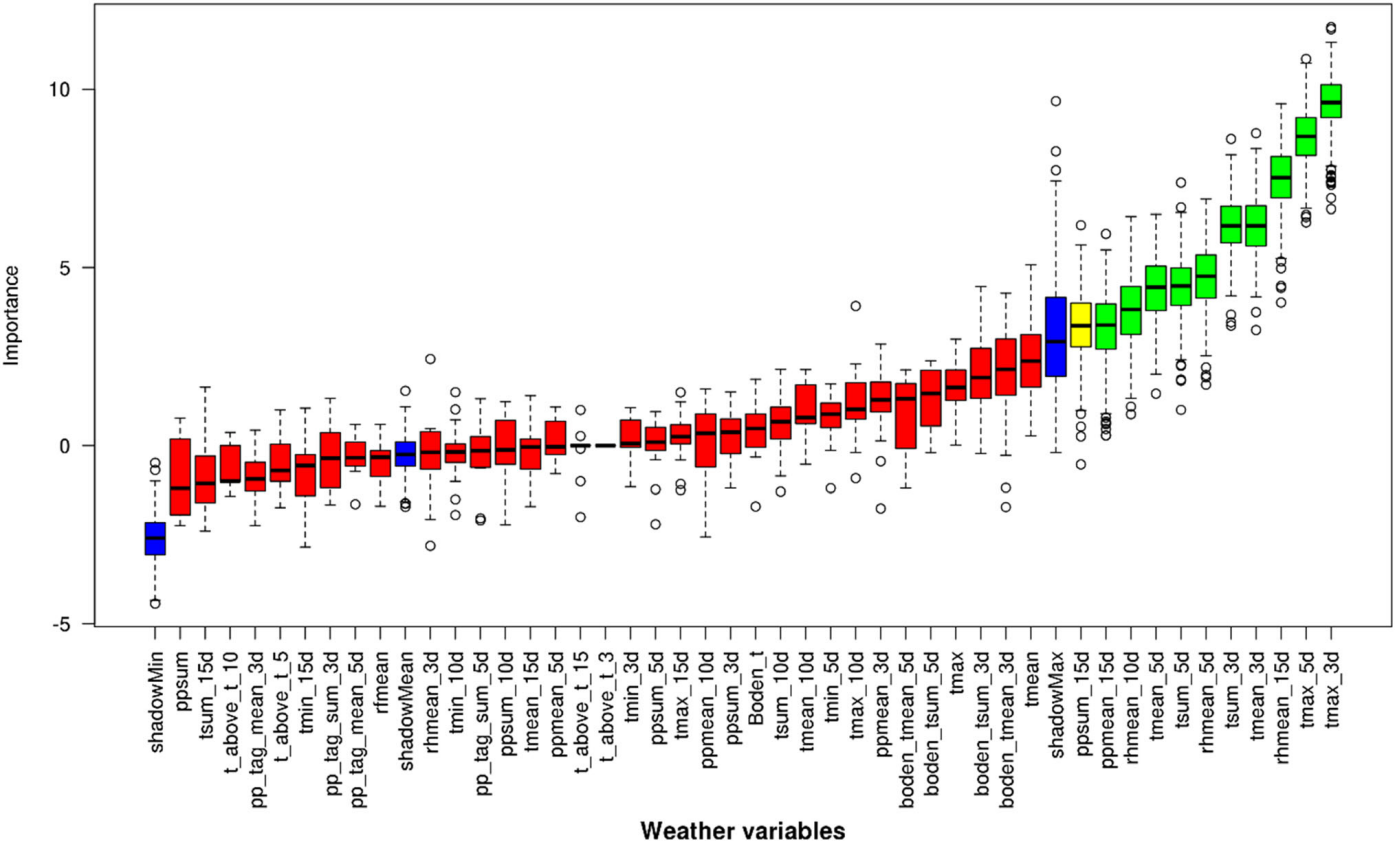
Classification of the weather variables by the Boruta algorithm. The variables are ranked from left to right in increasing order of importance. The boxplots represent 500 simulations. The three importance categories, confirmed, tentative and rejected are represented by the green, yellow and red boxes, respectively. The blue boxes represent the shadow parameters created by the model to check variable importance. All weather variables are presented in Table 1.

**Table 6:** Confusion matrix based on the prediction results of the sclerotia germination model, absolute and percentage values in brackets

		Predicted	
		1	0
Observed	1	22 (46.8%)	4 (8.5%)
	0	6 (12.8%)	15 (31.9%)

The total observations for sclerotia germination are 47, for one site in Germany for the years 2019–2023.

**Table 7:** Calibrated optimal values of temperature and relative humidity for Sclerotinia infection in winter rapeseed.

Parameters	Range	Source	Calibrated
Optimal temperature for infection (°C)	13–25	^10^, ^46^, ^47^	18
Optimal relative humidity for infection (%)	85–99	^10^, ^48^	90

### 3.3 Validation of the improved SkleroPro

Of the 59 units assessed across 37 site‐years (2019–2024), ten showed DI above 20%, 32 had no incidence, and 17 recorded DI between 1% and 20% (Table S1). Disease pressure peaked in 2022, with nearly 40% of units exceeding the 20% DI threshold, whereas in 2020 no units surpassed this level.

The improved SkleroPro model predicted Sclerotinia infection using a DI threshold of 20%, achieving an overall accuracy of 66% for the period 2020–2024, compared to 39% for the current SkleroPro model (Table 8 and Fig. 9). The enhanced version reduced the overestimations from 58% to 32% and the underestimation from 4% to 2%. The model correctly identified infection cases with a sensitivity (True Positive Rate) of 90%, while specificity (True Negative Rate) was 61% (Table 9). Overestimation of infection occurred in 32% of the sites, while underestimation (False Negative Rate) was observed in only one site in 2021. The model prediction results for units across all site‐years are presented in Table S2. A comparison between the current, the improved SkleroPro (with the CBD model) and the version keeping the Simonto‐WR model is presented in Table S3.

**Figure 9:**
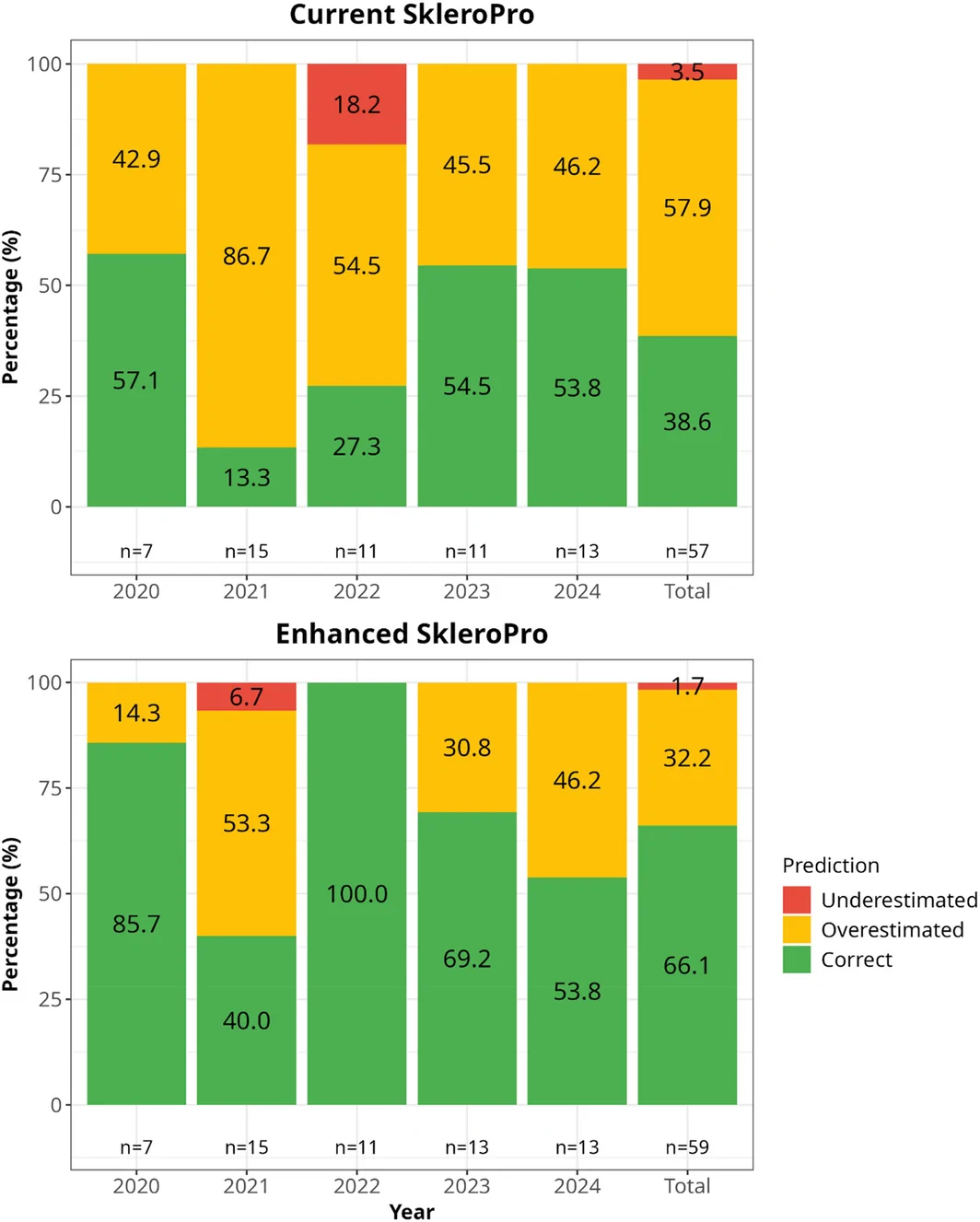
Validation results of the current (top) and improved (bottom) SkleroPro for the period 2020–2024. The *x*‐axis represents the years. The *y*‐axis shows the percentages of prediction according to three categories: underestimated (red), overestimated (yellow) and correct (green). Value *n* represents the total number of units used for validation.

**Table 8:** Comparison of model predictions (%) between the current and the enhanced SkleroPro for each year

	SkleroPro (current)		SkleroPro (enhanced)	
Year	Number of sites	Correct prediction (%)	Number of sites	Correct prediction (%)
2020	7	57	7	86
2021	15	13	15	40
2022	11	27	11	100
2023	11	55	13	69
2024	13	54	13	54
Total	57	39	59	66

The results comprise 37 site‐years and 59 units (site‐cultivar) for 5 years (2019–2024) in Germany.

**Table 9:** Confusion matrix of the prediction results of the improved SkleroPro, absolute and percentage values in brackets

		Predicted	
		1	0
Observed	1	9 (15.3%)	1 (1.7%)
	0	19 (32.2%)	30 (50.8%)

The results comprise 37 site‐years and 59 units (site‐cultivar) for 5 years (2019–2024) in Germany.

## 4 DISCUSSION

In this study, we developed a phenology model for winter rapeseed that predicts flowering stages starting from 1 February. The model demonstrated high predictive accuracy with an RMSE of 3.83 days and an *R* ^2^ of 90%, outperforming several existing models.^2^, ^3^, ^21^, ^25^ Validated over 5 years across a range of environmental conditions and 60 cultivars in Germany, it proved broadly applicable. However, inaccuracies around BBCH stage 58, which triggers the SkleroPro risk prediction model for Sclerotinia stem rot, resulted in a ±4‐day uncertainty window (Fig. 6). This may lead to premature fungicide applications or missed treatment windows, affecting disease control. While CBD and Simonto‐WR models showed comparable accuracy, our model's advantage lies in usability by eliminating the need for BBCH 55 input, which in the case of Simonto‐WR may be impractical due to time, labor, or access constraints.

Despite its general applicability, the CBD model's uncertainty arises from genetic diversity among cultivars, which may vary in their development rates.

A notable advancement in our study is the integration of a sclerotia germination model into SkleroPro. The previous version only assessed infection conditions, whereas our improved version first verifies whether sclerotia have germinated and ascospores are available for infection. The sclerotia germination prediction achieved an accuracy of nearly 80%, although this accuracy is based on internal cross‐validation and further independent validation is required. Predictors for the germination model – mean maximum temperature over 3 days and mean relative humidity over 15 days – capture key environmental conditions that influence germination. Short‐term warming promotes metabolic activity and apothecia development, while longer humidity periods sustain moisture for successful germination.^6^, ^11^, ^49^ The use of relative humidity is supported by existing literature demonstrating its strong influence on apothecial development and ascospore discharge,^6^, ^7^, ^19^, ^45^ and can be viewed as an integrative variable representative of moisture in the canopy microclimate.^31^ Although sclerotial germination may begin prior to full canopy closure, in practice, germination and subsequent apothecia and ascospore production are commonly observed during the flowering stage, aligning with increasing precipitation and rising soil temperatures.

Integrating this germination model into SkleroPro improved disease risk prediction. Between 2020 and 2024, the model increased the accuracy of the previous version from 39% to 66% (Table 8 and Fig. 9). Since CBD and Simonto‐WR models performed similarly, the improvement is primarily due to the inclusion of the germination and ascospore availability module. While this improvement is significant, accuracy remains moderate and varied across years, with particularly low performance in 2024. The high rate of overestimations in 2024 was likely due to an unusually early flowering period in Germany caused by warm March temperatures, followed by a sudden temperature drop in April. Although late April conditions favored Sclerotinia development, the early flowering caused the flowering period to conclude before optimal infection conditions were met, leading to a temporal mismatch between the phenology and epidemiology modules. Importantly, the model exhibited high sensitivity (90%), with only one missed outbreak over 5 years. This ‘risk‐averse’ behavior ensures that nearly all significant Sclerotinia outbreaks are correctly identified, which is critical for effective disease control. Similar to other forecasting tools, such as the one developed by Young *et al*.^50^ for the United Kingdom, the model overpredicts high‐risk scenarios as a precautionary measure, leading to a high number of false positives where fungicide treatments are recommended despite low actual infection risk. Low sclerotial density is an unlikely explanation for overprediction, as the study fields had a documented history of winter rapeseed cultivation and Sclerotinia outbreaks, indicating that sufficient inoculum was present for disease development. The 20% DI threshold used in this study refers specifically to Sclerotinia infections on the main stem, which are typically associated with significant yield losses. This DI threshold was also applied in the model developed by Shahoveisi and del Río Mendoza^51^ to assess Sclerotinia incidence in winter rapeseed in North Dakota.

The tendency to overpredict high‐risk scenarios can be attributed to the complex interplay of environmental factors influencing Sclerotinia stem rot development. Disease forecasting models rely on biological and meteorological variables like temperature, soil moisture, relative humidity and photoperiod,^9^, ^52^ which interact to influence crop phenology and the life cycle of *S. sclerotiorum*.^35^ Warm and moist conditions promote apothecia formation and ascospore release, while dry or cool conditions inhibit these processes.^30^ Even when weather conditions are suitable for infection, microclimatic and structural barriers – like canopy dryness or insufficient leaf wetness – may prevent infection,^6^ leading to further risk overestimation. Although soil moisture is crucial for sclerotia germination, modeling it at scale remains challenging. While some existing models incorporate soil moisture,^19^, ^31^ we developed a new model prioritizing practical use across large agricultural areas by incorporating widely available rainfall data to capture moisture effects. Although photoperiod is relatively stable, cooler spring weather can extend flowering and increase susceptibility, whereas warm, dry conditions shorten the infection window.^53^, ^54^, ^55^ Our long‐term field monitoring confirmed that apothecia can still develop after flowering, though such post‐flowering infections tend to be less severe.^30^ While we simplify this complexity by focusing on temperature and humidity, the model performs well across varied conditions, balancing biological realism and operational simplicity for practical use in decision‐support tools.

Despite improvements in modeling Sclerotinia disease risk, challenges remain. Regional variability in environmental conditions – such as air and soil temperature, humidity and rainfall – introduces uncertainty, making it difficult to develop a universally applicable model. High‐risk conditions may be predicted, but disease may not develop due to poor synchrony with flowering or limited spore load, which may result from low sclerotial density in the soil.^19^ Our model incorporates flowering phenology to align infection risk with crop susceptibility, but some misalignment is still possible. Additionally, pathogen‐specific factors such as isolate aggressiveness may affect disease development.^56^, ^57^ Our previous studies^8^, ^30^ observed significant differences in infection severity among isolates, highlighting the importance of isolate virulence. The current model is tailored for winter rapeseed, which flowers from April to May in Germany – typically aligning with favorable infection conditions. Applying the model to spring oilseed rape, which flowers later under drier and warmer conditions, would require further validation due to differing phenological and climatic dynamics. Lastly, predictions over the course of years may also be affected by the crop's and pathogen's responses to climate change, potentially altering their current optimal conditions and limiting thresholds.^35^

Several strategies could improve the SkleroPro model's accuracy and applicability. While empirical models are simple and user‐friendly, they may oversimplify biological interactions. We view the current version of SkleroPro as a step toward integrating mechanistic or hybrid models that better capture host–pathogen dynamics. Incorporating inoculum monitoring – such as spore trapping or petal testing – could improve the model's ability to distinguish between theoretical and actual infection risk.^6^, ^50^, ^58^ While most petals carry viable ascospores after release, infection depends on environmental conditions and where petals land. Petal testing, combined with environmental monitoring, can therefore improve disease risk forecasting.

## 5 CONCLUSION

The enhanced SkleroPro model represents a notable advancement in sustainable disease management for winter rapeseed, enabling identification of high‐ and low‐risk periods to guide fungicide application decisions. Integration of phenology and sclerotia germination components has improved prediction accuracy across diverse conditions. While around 30% of cases are still overestimated, further refinements could enhance specificity and user acceptance. The model's accessible inputs and high sensitivity support effective disease control. Following a testing phase with the German Plant Protection Service, it will be made freely available to farmers across Germany. Although SkleroPro does not prescribe specific spray schedules, its dynamic risk assessments help determine when treatment is justified during the flowering period. Continued validation will be essential to maintain performance under changing climatic and agronomic conditions.

## CONFLICT OF INTEREST

The authors declare no conflicts of interest.

## Supporting information


**Figure S1.** Observed BBCH stages (*y*‐axis) plotted against simulated cumulative biological days (CBD; *x*‐axis) for 142 sites from the training dataset. Each colored line represents one site. The thick black line shows the fitted double Gompertz curve capturing the general phenological trend across sites. The vertical red dashed line at CBD = 22.5 marks the threshold at which the second Gompertz function begins.
**Figure S2**. Relationship between sowing date and BBCH 55 date across 81 locations (2019–2023) in Germany. The *x*‐axis represents the day of the year (DOY) of sowing, while the *y*‐axis represents the DOY of BBCH 55 – the stage at which individual flowers of the main inflorescence become visible (closed). The scatter plot includes a linear regression line with a confidence interval, the Pearson correlation coefficient and the *P* value. Each point represents a specific location.
**Table S1**. Complete list of sites of the field trials and their corresponding Sclerotinia disease incidence, resulting in a total of 37 site‐years and 59 units for 5 years (2020–2024) in Germany. NA, not available data.
**Table S2**. Results of the enhanced SkleroPro model for all field trial units (*n* = 59) across 37 locations over five seasons (2020–2024) in Germany. The table reports observed Sclerotinia disease incidence, along with model predictions categorized as correct, overestimated, or underestimated. It indicates whether the model threshold was met, and if so, the corresponding date. NA, not available data.
**Table S3**. Comparison of predictions from the current SkleroPro, the enhanced SkleroPro, and the enhanced SkleroPro incorporating the Simonto‐WR phenology model for 20 sites in 2023 and 2024. The table includes observed Sclerotinia disease incidence alongside model predictions, categorized as correct, overestimated, or underestimated. Also shown are the predicted spore availability dates from the enhanced SkleroPro, as well as the flowering start and end dates as estimated by both the Simonto‐WR and CBD phenology models. Sites where the enhanced SkleroPro provided more accurate predictions than the current version are highlighted in bold.

## Data Availability

The data that support the findings of this study are available on request from the corresponding author. The data are not publicly available due to privacy or ethical restrictions.
