# Supplementary material for: Advancing Sclerotinia risk forecasting for winter rapeseed in Germany: integrating crop phenology and disease development into a decision support system
Source: Pest Manag Sci. 2025 Aug 22;82(11):10828–40. doi: 10.1002/ps.70166 (PMC13665595; doi:10.1002/ps.70166)
Supplement: Supplementary file 1 [file PS-82-10828-s001.docx]

Supplementary material

Figure S1. Observed BBCH stages (y-axis) plotted against simulated cumulative biological days (CBD; x-axis) for 142 sites from the training dataset. Each colored line represents one site. The thick black line shows the fitted double Gompertz curve capturing the general phenological trend across sites. The vertical red dashed line at CBD = 22.5 marks the threshold at which the second Gompertz function begins.

Figure S2. Relationship between sowing date and BBCH 55 date across 81 locations (2019–2023) in Germany. The x-axis represents the day of the year (DOY) of sowing, while the y-axis represents the DOY of BBCH 55—the stage at which individual flowers of the main inflorescence become visible (closed). The scatter plot includes a linear regression line with a confidence interval, the Pearson correlation coefficient and the p value. Each point represents a specific location.

Table S1: Complete list of sites of the field trials and their corresponding Sclerotinia disease incidence, resulting in a total of 37 site-years and 59 units for five years (2020-2024) in Germany. NA= Not available data

| Federal State | Location | Year | Winter rapeseed cultivar | Frequency of winter rapeseed rotation (years) | Sclerotinia Disease Incidence (%) |
| --- | --- | --- | --- | --- | --- |
| Baden-Württemberg | Renningen | 2020 | NA | 3 | 0 |
| Baden-Württemberg | Renningen | 2021 | Avatar | 3 | 14 |
| Baden-Württemberg | Renningen | 2021 | Armani | 3 | 21 |
| Baden-Württemberg | Renningen | 2022 | Avatar | 3 | 0 |
| Baden-Württemberg | Renningen | 2022 | Armani | 3 | 0 |
| Baden-Württemberg | Renningen | 2023 | Avatar | 2 | 0 |
| Baden-Württemberg | Renningen | 2023 | Bender | 2 | 0 |
| Bavaria | Puch | 2020 | NA | 3 | 0 |
| Bavaria | Puch | 2021 | Avatar | 3 | 0 |
| Bavaria | Puch | 2021 | Christiano | 3 | 0 |
| Bavaria | Puch | 2022 | Avatar | 3 | 0 |
| Bavaria | Puch | 2022 | Ernesto | 3 | 0 |
| Bavaria | Puch | 2023 | Avatar | 3 | 0 |
| Bavaria | Puch | 2023 | Ernesto | 3 | 0 |
| Bavaria | Puch | 2024 | Avatar | 6 | 0 |
| Bavaria | Puch | 2024 | Ernesto | 6 | 0 |
| Brandenburg | Danewitz | 2020 | NA | 2 | 5 |
| Brandenburg | Frankfurtoder | 2020 | NA | 2 | 0 |
| Brandenburg | Ellingen | 2021 | RGT Trezzor | 4 | 11.5 |
| Brandenburg | Juteborg | 2021 | Eraton | 3 | 9.5 |
| Brandenburg | Nuhnen | 2021 | PT271 | 2 | 18.5 |
| Brandenburg | Nuhnen | 2022 | NA | 3 | 0 |
| Brandenburg | Tempelfelde | 2023 | NA | 2 | 6 |
| Brandenburg | Nuhnen | 2024 | PT303 | 3 | 0 |
| Brandenburg | Nuhnen | 2024 | Ambassador | 3 | 0 |
| Brandenburg | Tempelfelde | 2024 | NA | 3 | 0 |
| Mecklenburg-Vorpommern | Goddin | 2020 | NA | 3 | 8 |
| Mecklenburg-Vorpommern | Gross_Kiesow | 2020 | NA | 4 | 3 |
| Mecklenburg-Vorpommern | Rostock_Biestow | 2020 | NA | 2 | 12 |
| Mecklenburg-Vorpommern | Biestow | 2021 | Avatar | 2 | 7 |
| Mecklenburg-Vorpommern | Biestow | 2021 | Violin | 2 | 8 |
| Mecklenburg-Vorpommern | Gulzow | 2021 | Violin | 2 | 3 |
| Mecklenburg-Vorpommern | Biestow | 2022 | DK Expansion | 2 | 41 |
| Mecklenburg-Vorpommern | Biestow | 2022 | Avatar | 2 | 45 |
| Mecklenburg-Vorpommern | Biestow | 2023 | Avatar | 2 | 0 |
| Mecklenburg-Vorpommern | Biestow | 2023 | Bender | 2 | 0 |
| Mecklenburg-Vorpommern | Biestow | 2024 | PT303 | 2 | 0 |
| Mecklenburg-Vorpommern | Biestow | 2024 | Avatar | 2 | 0 |
| North Rhine-Westphalia | Schwefe | 2021 | Alvaro | 4 | 35 |
| Saxony-Anhalt | Siedengrieben | 2021 | Avatar | 4 | 1 |
| Saxony-Anhalt | Siedengrieben | 2021 | Alvaro | 4 | 2 |
| Saxony-Anhalt | Beetzendorf | 2022 | Alvaro | 3 | 2 |
| Saxony-Anhalt | Beetzendorf | 2022 | Avatar | 3 | 10 |
| Saxony-Anhalt | Beetzendorf | 2023 | Avatar | 3 | 0 |
| Saxony-Anhalt | Beetzendorf | 2023 | Bender | 3 | 0 |
| Saxony-Anhalt | Beetzendorf | 2024 | Avatar | 2 | 1 |
| Saxony-Anhalt | Beetzendorf | 2024 | Heiner | 2 | 0 |
| Schleswig-Holstein | Futterkamp | 2021 | Avatar | 4 | 0 |
| Schleswig-Holstein | Futterkamp | 2021 | Armani | 4 | 0 |
| Schleswig-Holstein | Oesterdeichstrich | 2022 | Armani | 2 | 39 |
| Schleswig-Holstein | Oesterdeichstrich | 2022 | Avatar | 2 | 52 |
| Schleswig-Holstein | Futterkamp | 2023 | Avatar | 4 | 0 |
| Schleswig-Holstein | Futterkamp | 2023 | Bender | 4 | 0 |
| Schleswig-Holstein | Oesterdeichstrich | 2023 | Bender | 2 | 20 |
| Schleswig-Holstein | Oesterdeichstrich | 2023 | Avatar | 2 | 23 |
| Schleswig-Holstein | Futterkamp | 2024 | Avatar | 5 | 0 |
| Schleswig-Holstein | Futterkamp | 2024 | Heiner | 1 | 0 |
| Schleswig-Holstein | Hedwigenkoog | 2024 | Avatar | 1 | 90 |
| Schleswig-Holstein | Hedwigenkoog | 2024 | Heiner | 1 | 59 |

Table S2: Results of the enhanced SkleroPro model for all field trial units (n = 59) across 37 locations over five seasons (2020–2024) in Germany. The table reports observed Sclerotinia disease incidence, along with model predictions categorized as correct, overestimated, or underestimated. It indicates whether the model threshold was met, and if so, the corresponding date. NA= Not available data.

| Federal State | Location | Year | Winter rapeseed cultivar | Frequency of winter rapeseed rotation (years) | Sclerotinia Disease Incidence (%) | Enhanced SkleroPro threshold exceeded | Threshold reached (date) | Enhanced SkleroPro prediction |
| --- | --- | --- | --- | --- | --- | --- | --- | --- |
| Baden-Württemberg | Renningen | 2020 | NA | 3 | 0 | No |  | Correct |
| Bavaria | Puch | 2020 | NA | 3 | 0 | No |  | Correct |
| Brandenburg | Danewitz | 2020 | NA | 2 | 5 | No |  | Correct |
| Brandenburg | Frankfurtoder | 2020 | NA | 3 | 0 | No |  | Correct |
| Mecklenburg-Vorpommern | Goddin | 2020 | NA | 3 | 8 | No |  | Correct |
| Mecklenburg-Vorpommern | Gross_Kiesow | 2020 | NA | 4 | 3 | No |  | Correct |
| Mecklenburg-Vorpommern | Rostock_Biestow | 2020 | NA | 2 | 12 | Yes | 18.05.2020 | Overestimated |
| Baden-Württemberg | Renningen | 2021 | Avatar | 3 | 14 | No |  | Correct |
| Baden-Württemberg | Renningen | 2021 | Armani | 3 | 21 | No |  | Underestimated |
| Bavaria | Puch | 2021 | Avatar | 3 | 0 | No |  | Correct |
| Bavaria | Puch | 2021 | Christiano | 3 | 0 | No |  | Correct |
| Brandenburg | Ellingen | 2021 | RGT Trezzor | 4 | 11.5 | Yes | 13.05.2021 | Overestimated |
| Brandenburg | Juteborg | 2021 | Eraton | 3 | 9.5 | Yes | 12.05.2021 | Overestimated |
| Brandenburg | Nuhnen | 2021 | PT271 | 2 | 18.5 | Yes | 12.05.2021 | Overestimated |
| Mecklenburg-Vorpommern | Biestow | 2021 | Avatar | 2 | 7 | Yes | 12.05.2021 | Overestimated |
| Mecklenburg-Vorpommern | Biestow | 2021 | Violin | 2 | 8 | Yes | 12.05.2021 | Overestimated |
| Mecklenburg-Vorpommern | Gulzow | 2021 | Violin | 2 | 3 | Yes | 12.05.2021 | Overestimated |
| North Rhine-Westphalia | Schwefe | 2021 | Alvaro | 4 | 35 | Yes | 12.05.2021 | Correct |
| Saxony-Anhalt | Siedengrieben | 2021 | Avatar | 4 | 1 | Yes | 12.05.2021 | Overestimated |
| Saxony-Anhalt | Siedengrieben | 2021 | Alvaro | 4 | 2 | Yes | 12.05.2021 | Overestimated |
| Schleswig-Holstein | Futterkamp | 2021 | Avatar | 4 | 0 | No |  | Correct |
| Schleswig-Holstein | Futterkamp | 2021 | Armani | 4 | 0 | No |  | Correct |
| Baden-Württemberg | Renningen | 2022 | Avatar | 3 | 0 | No |  | Correct |
| Baden-Württemberg | Renningen | 2022 | Armani | 3 | 0 | No |  | Correct |
| Bavaria | Puch | 2022 | Avatar | 3 | 0 | No |  | Correct |
| Bavaria | Puch | 2022 | Ernesto | 3 | 0 | No |  | Correct |
| Brandenburg | Nuhnen | 2022 | NA | 3 | 0 | No |  | Correct |
| Mecklenburg-Vorpommern | Biestow | 2022 | DK Expansion | 2 | 41 | Yes | 21.05.2022 | Correct |
| Mecklenburg-Vorpommern | Biestow | 2022 | Avatar | 2 | 45 | Yes | 21.05.2022 | Correct |
| Saxony-Anhalt | Beetzendorf | 2022 | Alvaro | 3 | 2 | No |  | Correct |
| Saxony-Anhalt | Beetzendorf | 2022 | Avatar | 3 | 10 | No |  | Correct |
| Schleswig-Holstein | Oesterdeichstrich | 2022 | Armani | 2 | 39 | Yes | 11.05.2022 | Correct |
| Schleswig-Holstein | Oesterdeichstrich | 2022 | Avatar | 2 | 52 | Yes | 11.05.2022 | Correct |
| Baden-Württemberg | Renningen | 2023 | Avatar | 2 | 0 | Yes | 08.05.2023 | Overestimated |
| Baden-Württemberg | Renningen | 2023 | Bender | 2 | 0 | Yes | 08.05.2023 | Overestimated |
| Bavaria | Puch | 2023 | Avatar | 3 | 0 | Yes | 08.05.2023 | Overestimated |
| Bavaria | Puch | 2023 | Ernesto | 3 | 0 | Yes | 08.05.2023 | Overestimated |
| Brandenburg | Tempelfelde | 2023 | NA | 2 | 6 | No |  | Correct |
| Mecklenburg-Vorpommern | Biestow | 2023 | Avatar | 2 | 0 | No |  | Correct |
| Mecklenburg-Vorpommern | Biestow | 2023 | Bender | 2 | 0 | No |  | Correct |
| Saxony-Anhalt | Beetzendorf | 2023 | Avatar | 3 | 0 | No |  | Correct |
| Saxony-Anhalt | Beetzendorf | 2023 | Bender | 3 | 0 | No |  | Correct |
| Schleswig-Holstein | Futterkamp | 2023 | Avatar | 4 | 0 | No |  | Correct |
| Schleswig-Holstein | Futterkamp | 2023 | Bender | 4 | 0 | No |  | Correct |
| Schleswig-Holstein | Oesterdeichstrich | 2023 | Bender | 2 | 20 | Yes | 10.05.2023 | Correct |
| Schleswig-Holstein | Oesterdeichstrich | 2023 | Avatar | 2 | 23 | Yes | 10.05.2023 | Correct |
| Bavaria | Puch | 2024 | Avatar | 6 | 0 | No |  | Correct |
| Bavaria | Puch | 2024 | Ernesto | 6 | 0 | No |  | Correct |
| Brandenburg | Nuhnen | 2024 | PT 303 | 3 | 0 | No |  | Correct |
| Brandenburg | Nuhnen | 2024 | Ambassador | 3 | 0 | No |  | Correct |
| Brandenburg | Tempelfelde | 2024 | NA | 3 | 0 | No |  | Correct |
| Mecklenburg-Vorpommern | Biestow | 2024 | PT 303 | 2 | 0 | Yes | 06.04.2024 | Overestimated |
| Mecklenburg-Vorpommern | Biestow | 2024 | Avatar | 2 | 0 | Yes | 06.04.2024 | Overestimated |
| Saxony-Anhalt | Beetzendorf | 2024 | Avatar | 2 | 1 | Yes | 05.04.2024 | Overestimated |
| Saxony-Anhalt | Beetzendorf | 2024 | Heiner | 2 | 0 | Yes | 05.04.2024 | Overestimated |
| Schleswig-Holstein | Futterkamp | 2024 | Avatar | 5 | 0 | Yes | 12.04.2024 | Overestimated |
| Schleswig-Holstein | Futterkamp | 2024 | Heiner | 5 | 0 | Yes | 12.04.2024 | Overestimated |
| Schleswig-Holstein | Hedwigenkoog | 2024 | Avatar | 1 | 90 | Yes | 06.04.2024 | Correct |
| Schleswig-Holstein | Hedwigenkoog | 2024 | Heiner | 1 | 59 | Yes | 06.04.2024 | Correct |

Table S3: Comparison of predictions from the current SkleroPro, the enhanced SkleroPro, and the enhanced SkleroPro incorporating the Simonto-WR phenology model for 20 sites in 2023 and 2024. The table includes observed Sclerotinia disease incidence alongside model predictions, categorized as correct, overestimated, or underestimated. Also shown are the predicted spore availability dates from the enhanced SkleroPro, as well as the flowering start and end dates as estimated by both the Simonto-WR and CBD phenology models. Sites where the enhanced SkleroPro provided more accurate predictions than the current version are highlighted in bold.

| Site ID | Year | Sclerotinia Disease Incidence (%) | Current SkleroPro | Enhanced SkleroPro | Enhanced SkleroPro with Simonto-WR model | Spore availability window (April/May) | BBCH 58 Simonto | BBCH 58 CBD | BBCH 70 Simonto | BBCH 70 CBD |
| --- | --- | --- | --- | --- | --- | --- | --- | --- | --- | --- |
| **BB_Tempelfelde** | **2023** | **6** | **overestimated** | **correct** | **correct** | **no spores from 15 May** | **03.05.2023** | **20.04.2023** | **26.05.2023** | **21.05.2023** |
| SH_Futterkamp Avatar | 2023 | 0 | correct | correct | correct | no spores 18 May | 22.04.2023 | 17.04.2023 | 26.05.2023 | 22.05.2023 |
| SH_Futterkamp Bender | 2023 | 0 | correct | correct | correct | no spores 18 May | 24.04.2023 | 17.04.2023 | 27.05.2023 | 22.05.2023 |
| BY_Puch_Avatar | 2023 | 0 | overestimated | overestimated | overestimated | all flowering period | 21.04.2023 | 19.04.2023 | 24.05.2023 | 22.05.2023 |
| BY_Puch_Ernesto | 2023 | 0 | overestimated | overestimated | overestimated | all flowering period | 21.04.2023 | 19.04.2023 | 24.05.2023 | 22.05.2023 |
| **MV_Biestow_Avatar** | **2023** | **0** | **overestimated** | **correct** | **correct** | **no spores 16-17 May** | **22.04.2023** | **21.04.2023** | **25.05.2023** | **24.05.2023** |
| **MV_Biestow_Bender** | **2023** | **0** | **overestimated** | **correct** | **correct** | **no spores 16-17 May** | **22.04.2023** | **21.04.2023** | **25.05.2023** | **24.05.2023** |
| BB_Nuhnen_PT303 | 2024 | 0 | correct | correct | correct | no spores from 07 May | 01.04.2024 | 28.03.2024 | 02.05.2024 | 01.05.2024 |
| BB_Nuhnen_Ambassador | 2024 | 0 | correct | correct | correct | no spores from 07 May | 01.04.2024 | 28.03.2024 | 02.05.2024 | 01.05.2024 |
| BB_Tempelfelde | 2024 | 0 | correct | correct | correct | no spores from 07 May | 31.03.2024 | 29.03.2024 | 02.05.2024 | 02.05.2024 |
| BY_Puch_Avatar | 2024 | 0 | correct | correct | correct | all flowering period | 30.03.2024 | 29.03.2024 | 03.05.2024 | 04.05.2024 |
| BY_Puch_Ernesto | 2024 | 0 | correct | correct | correct | all flowering period | 30.03.2024 | 29.03.2024 | 03.05.2024 | 04.05.2024 |
| ST_Beetzendorf_Avatar | 2024 | 0 | overestimated | overestimated | overestimated | no spores 15-29 May | 31.03.2024 | 27.03.2024 | 03.05.2024 | 30.04.2024 |
| ST_Beetzendorf_Heiner | 2024 | 0 | overestimated | overestimated | overestimated | no spores 15-29 May | 31.03.2024 | 27.03.2024 | 03.05.2024 | 30.04.2024 |
| MV_Biestow_PT303 | 2024 | 0 | overestimated | overestimated | overestimated | all flowering period | 01.04.2024 | 02.04.2024 | 08.05.2024 | 08.05.2024 |
| MV_Biestow_Avatar | 2024 | 0 | overestimated | overestimated | overestimated | all flowering period | 01.04.2024 | 02.04.2024 | 08.05.2024 | 08.05.2024 |
| SH_Futterkamp_Avatar | 2024 | 0 | overestimated | overestimated | overestimated | all flowering period | 30.03.2024 | 01.04.2024 | 09.05.2024 | 08.05.2024 |
| SH_Futterkamp_Heiner | 2024 | 0 | overestimated | overestimated | overestimated | all flowering period | 30.03.2024 | 01.04.2024 | 09.05.2024 | 08.05.2024 |
| SH_Hedwigenkoog_Avatar | 2024 | 90 | correct | correct | correct | all flowering period | 06.04.2024 | 30.03.2024 | 14.05.2024 | 15.05.2024 |
| SH_Hedwigenkoog_Heiner | 2024 | 59 | correct | correct | correct | all flowering period | 06.04.2024 | 30.03.2024 | 14.05.2024 | 15.05.2024 |
